# A Study on the Mechanical Properties of an Automobile Part Additively Printed through Periodic Layer Rotation Strategies

**DOI:** 10.3390/ma15010070

**Published:** 2021-12-22

**Authors:** Min-Seok Yang, Ji-Heon Kang, Ji-Wook Kim, Kun-Woo Kim, Da-Hye Kim, Ji-Hyun Sung, Dae-Cheol Ko, Jae-Wook Lee

**Affiliations:** 1 Dae-Gyeong Division, Korea Institute of Industrial Technology, Daegu 42994, Korea; msyang@kitech.re.kr (M.-S.Y.); kangji1226@kitech.re.kr (J.-H.K.); jwkim0@kitech.re.kr (J.-W.K.); kwkim@kitech.re.kr (K.-W.K.); dahye.kim@kitech.re.kr (D.-H.K.); jsung@kitech.re.kr (J.-H.S.); 2 Department of Nanomechatronics Engineering, Pusan National University, Pusan 46241, Korea

**Keywords:** additive manufacturing, periodic layer rotation, finite elements analysis, scan strategy, isotropy verification

## Abstract

In metal product manufacturing, additive manufacturing (AM) is a method that has the advantage of fabricating complex shapes and customized production, unlike existing machining methods. However, owing to the characteristics of the AM process, anisotropy of macrostructure occurs because of various causes such as the scan direction, melting, fusion, and cooling of the powdered material. The macrostructure anisotropy is realized from the scan direction, and when a single layer is stacked in one direction, it is expressed as orthogonal anisotropy. Here, the classical lamination theory is applied to simply calculate the individual orthotropic layers by superimposing them. Through this, the authors analyzed whether the mechanical properties of the product are isotropically expressed with a periodic layer rotation strategy. To determine if the mechanical properties can be reasonably considered to be isotropic, a shock absorber mount for a vehicle was manufactured by AM. The tensile and vibration test performed on the product was compared with the finite element analysis and experimental results. As a result of the comparison, it was confirmed that the macroscopically of the product was considered isotropic as the load-displacement diagram and the fracture location coincided, as well as the natural frequency and mode shape.

## 1. Introduction

The basic concept of the additive manufacturing (AM) process is to produce the desired shape directly from 3D digital modeling data [1]. Unlike traditional machining processes, such as casting or cutting, the materials are stacked layer by layer to create products [1,2]. Because of this basic process difference, special measures (such as design for AM) in the design stage of additive products can resolve the disadvantages of the existing traditional manufacturing processes [2] and can easily control the AM process. Therefore, AM is attracting attention as a desirable manufacturing method for the design and production of high-performance components [1].

Metal AM has a high degree of freedom in molding, such as custom designs; however, problems in the mechanical properties of the product may occur because of the anisotropy of the structure due to the nature of the manufacturing process [3]. The DED process is additively manufactured by depositing in a local area [2,4], and the PBF process is a molding technique in which a flat powder area is melted and stacked with the selective thermal energy of a laser [2]. In particular, in the manufacture of products, the PBF process is widely used in the AM process as a representative example of metal AM [1]. The material has a thermal history during melting and solidification, and the solidified microstructure has directionality according to the movement path of the heat source. This affects the microstructure and mechanical properties of the alloys. As a major phenomenon, deformation by residual stress is induced, leading to distortion of or damage to the product. The directionality of the microstructure appears as an anisotropic property of the product. This problem causes dimensional inaccuracy of the product or affects the dynamic or static behavior of the structure, becoming another variable to be considered in the design stage. This acts as a constraint on the design and lowers the usefulness of metal AM with high formability.

To solve these problems, a periodic layer rotation strategy has been used to suppress the directionality of the material. Arısoy et al. stated that when the appropriate energy density and hatching angle were set to 67°, a fine grain size was obtained, and an isotropic structure was exhibited [5]. According to a study by Guan et al., fabricating a product with the PBF process using an appropriate hatch angle results in part exhibiting excellent tensile properties because it realizes microstructural isotropy and avoids stress concentration [6]. However, these studies were confirmed only through tensile tests at the specimen level.

In order to evaluate from a product level point of view, a study on mechanical properties evaluation considering macrostructure has also been conducted. Macrostructure characteristics were evaluated using the lamination process parameters similar to those of the AlSi10Mg material applied in this study. Thijs et al. The macrostructure of additive manufacturing materials revealed that morphological and crystallographic textures are realized from scanning strategies, which are expressed as anisotropy or isotropy [7]. Xiong et al. also confirmed the anisotropy of the macroscopic structure in the variable state of the additive manufacturing process similar to the application of AlSi10Mg material. In this study, it was shown that the crystal orientation of the melt pool boundary was dominant in determining the anisotropy of the material [8]. It is also explained that the scan direction has a significant influence on the anisotropy. It was proved that when the hatch angle is 90 degrees, the texture of the microstructure is further lowered, and the product is made isotropic. Furthermore, it was explained that if the product is laminated with a hatch angle other than 90 degrees, the texture of the product can be further lowered and made more isotropic. These studies commonly explain that the microscopic anisotropy of a material, which is expressed microscopically according to the heat flow characteristics in the additive manufacturing process, is only a small part, and the influence of the macroscopic structural characteristics obtained from the scan direction is more dominant.

The present study determined whether the anisotropic feature of the product generated along the laser scanning vector was removed using the periodic layer rotation strategy and expressed as isotropy. In Section 2, classical lamination theory was used to determine whether the AM part using the raster pattern strategy exhibited anisotropic properties when the mechanical properties of the product were inferred to be isotropic from the periodic layer rotation strategy. The verification of the basis of isotropy inferred from the evaluation of these numerical viewpoints was verified by a comparison with experiments and finite element (FE) analysis. Section 3 describes the design process of the vehicle shock absorber mount and shows the AM process of applying periodic layer rotation. In Section 4, tensile and modal tests performed on additive-manufactured products are described, and each product is verified through a comparison of the FE analysis to which isotropic properties are applied. Finally, Section 5 presents a discussion of the study results, and Section 6 presents the conclusion.

## 2. Evaluation of Mechanical Properties Using Classical Lamination Theory

This section evaluates the material properties of the products from a numerical perspective using the AM method of periodically rotating layers. The intrinsic strain method is widely used to perform additive process simulations [9]. This methodology is highly similar to the approach used in the classical lamination theory applied to characterize the behavior of composites [10,11]. The PBF process is also a layer-by-layer manufacturing process and has many similarities to the techniques used to produce composite laminates. In both cases, the final structure is layered, and anisotropy is induced. Here, the hatching sequence of the AM process produces the same effect as the fiber orientation of the composite [12]. Therefore, using the classical lamination theory, it is possible to infer the material properties at the product level. The strain tensor of the layer unit has a property that is orthogonal because of the hatching property, such as the fiber direction of the composite material. Additionally, the deformation component in the build direction (z=Z) becomes negligible as it is compensated by the re-coating step of the PBF process. Therefore, the strain at the layer level can be viewed as a plane stress state in the hatch direction (x) and its perpendicular direction (y) in the layer plane. Here, xyz is expressed in lowercase letters, as they represent the local coordinate system.

The hatching sequence of the metal AM process requires the following three assumptions in the classical lamination theory to simply analyze the problem of the composite laminate from the perspective of material mechanics [10,13,14]:

1. When the composite laminate was not subjected to bending, the strain in the thickness direction of the laminate was constant;
2. The strain on each ply in the plane was equal to the strain on the laminate;
3. The modulus of elasticity of each ply in the plane was different; thus, the stress had a different value for each ply.

**Figure 1:**
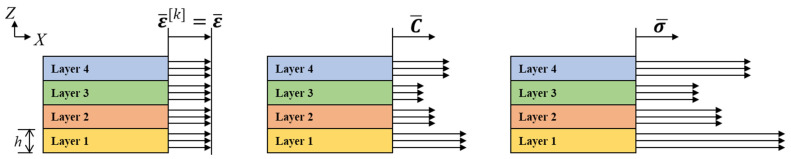
Schematic representation of Equation (1).

The three assumptions are expressed as Equation (1) by writing the stress of the $k^{th}$ layer (ply), which is a plane stress state, in the global coordinate system (XYZ coordinates) with the build direction as the axis.

(1) $${\bar{\sigma }}^{\left[k\right]}={\bar{C}}^{\left[k\right]}{\bar{\varepsilon }}^{\left[k\right]}$$

where ${\bar{\sigma }}^{\left[k\right]}$ is the stress component of the $k^{th}$ layer (ply) displayed with respect to the plane, ${\bar{C}}^{\left[k\right]}$ represents the elastic constitutive matrix of the material in the global coordinates, and ${\bar{\varepsilon }}^{\left[k\right]}$ represents the strain component of the $k^{th}$ layer (ply) displayed with respect to the layer plane. Additionally, the bar above the vector or matrix indicates that it is displayed in the global coordinate system. A schematic representation of Equation (1) is shown in Figure 1.

In the case of stacking with rotation for each layer and according to the classical lamination theory, the strain appears to be the same throughout the entire layer. That is, it is expressed as ${\bar{\varepsilon }}^{\left[k\right]}=\bar{\varepsilon }$. However, because the hatching directions of each layer are different, each stiffness matrix is expressed differently based on the global coordinates. Therefore, the stress is also different for each layer in the global coordinate system. Here, the sum of the stresses acting on a unit volume can be calculated by integrating the stress components of each layer. Furthermore, because the heights h of the layers in the unit volume are the same, the force per unit length $\bar{P}$ can be expressed as Equation (2):

(2) $$\bar{P}=\overset{N}{\underset{k=1}{\sum }}h^{\left[k\right]}{\bar{\sigma }}^{\left[k\right]}$$

where N is the number of layers per unit volume. Substituting Equation (1) into Equation (2) yields

(3) $$\bar{P}=\overset{N}{\underset{k=1}{\sum }}h^{\left[k\right]}{\bar{C}}^{\left[k\right]}{\bar{\varepsilon }}^{\left[k\right]}$$

Here, $\bar{P}$ is the amount integrated in the thickness direction; therefore, it becomes the force per unit length in the thickness direction of the layer. Therefore, stiffness $\bar{Q}$ is expressed as

(4) $$\bar{Q}=\overset{N}{\underset{k=1}{\sum }}h^{\left[k\right]}{\bar{C}}^{\left[k\right]}$$

Therefore, the force per unit length $\bar{P}$ is

(5) $$\bar{P}=\bar{Q}\bar{\varepsilon }$$

By considering the stress in the plane state, Equation (5) can be rewritten in matrix form as follows:

(6) $$\left\{\begin{array}{c} {\bar{P}}_{X}\\ {\bar{P}}_{Y}\\ {\bar{P}}_{XY} \end{array}\right\}=\left[\begin{array}{ccc} {\bar{Q}}_{11} & {\bar{Q}}_{12} & {\bar{Q}}_{16}\\ {\bar{Q}}_{12} & {\bar{Q}}_{22} & {\bar{Q}}_{26}\\ {\bar{Q}}_{16} & {\bar{Q}}_{26} & {\bar{Q}}_{66} \end{array}\right]\left\{\begin{array}{c} {\bar{\varepsilon }}_{X}\\ {\bar{\varepsilon }}_{Y}\\ {\bar{\gamma }}_{XY} \end{array}\right\}$$

Then, Equation (1) on the $k^{th}$ layer unit can be rewritten in matrix form as

(7) $${\left\{\begin{array}{c} {\bar{\sigma }}_{X}\\ {\bar{\sigma }}_{Y}\\ {\bar{\tau }}_{XY} \end{array}\right\}}^{\left[k\right]}={\left[\begin{array}{ccc} {\bar{C}}_{11} & {\bar{C}}_{12} & {\bar{C}}_{16}\\ {\bar{C}}_{12} & {\bar{C}}_{22} & {\bar{C}}_{26}\\ {\bar{C}}_{16} & {\bar{C}}_{26} & {\bar{C}}_{66} \end{array}\right]}^{\left[k\right]}{\left\{\begin{array}{c} {\bar{\varepsilon }}_{X}\\ {\bar{\varepsilon }}_{Y}\\ {\bar{\gamma }}_{XY} \end{array}\right\}}^{\left[k\right]}$$

**Figure 2:**
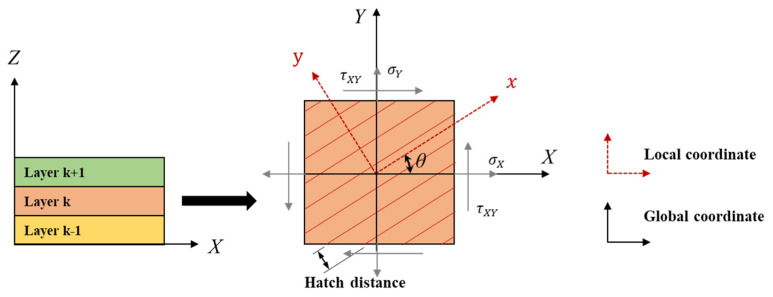
Schematic of the layer’s local coordinate system.

As shown in Figure 2, if the transformation and stiffness matrices of the local coordinate system are written before the coordinate transformation for each layer, the transformation can be expressed as follows:

(8) $$\sigma^{\left[k\right]}=\left[T_{1}\right]{\bar{\sigma }}^{\left[k\right]}=\left[\begin{array}{ccc} m^{2} & n^{2} & 2mn\\ n^{2} & m^{2} & -2mn\\ -mn & mn & m^{2}-n^{2} \end{array}\right]{\left\{\begin{array}{c} {\bar{\sigma }}_{X}\\ {\bar{\sigma }}_{Y}\\ {\bar{\tau }}_{XY} \end{array}\right\}}^{\left[k\right]}\;\varepsilon^{\left[k\right]}=\left[T_{2}\right]{\bar{\varepsilon }}^{\left[k\right]}=\left[\begin{array}{ccc} m^{2} & n^{2} & mn\\ n^{2} & m^{2} & -mn\\ -2mn & 2mn & m^{2}-n^{2} \end{array}\right]{\left\{\begin{array}{c} {\bar{\varepsilon }}_{X}\\ {\bar{\varepsilon }}_{Y}\\ {\bar{\gamma }}_{XY} \end{array}\right\}}^{\left[k\right]}$$

Here, m is cosθ, and n is sinθ, and $\left[T_{1}\right]$ and $\left[T_{2}\right]$ are the coordinate transformation matrices, respectively. Therefore, if the stress-strain relationship in the plane stress state is written as a local coordinate system, it can be expressed as

(9) $$\sigma^{\left[k\right]}=C^{\left[k\right]}\varepsilon^{\left[k\right]}$$

Using the transformation matrix of Equation (8), the elastic constitutive matrix ${\bar{C}}^{\left[k\right]}$, of the $k^{th}$ layer can be expressed as:

(10) $${\bar{C}}^{\left[k\right]}={\left[T_{1}\right]}^{-1}C^{\left[k\right]}\left[T_{2}\right]$$

**Figure 3:**
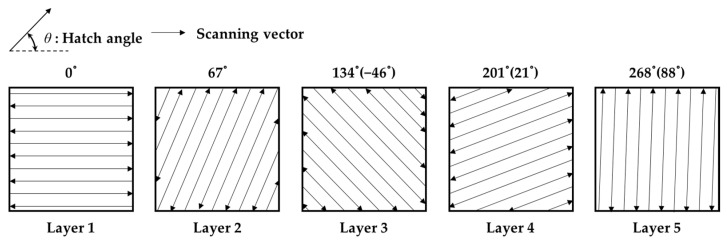
Schematic of 67° rotation increments of a layer using a raster scanning strategy.

Figure 3 shows the scanning vector up to the fifth layer when the hatch angle is 67°. The scanning vectors follow a raster pattern. It can be observed that the subsequent vector is rotated by 180° to change the direction. Here, the maximum hatch angle is expressed as 90° and above 180°, which is the same as the −θ value. If only integers are considered for the hatch angles, in the case of the hatch angles of prime numbers that do not have a common divisor with 180, and if 180 layers are stacked, all angles from 0° to 179° can be expressed. Therefore, considering the case of a unit volume expressed by all hatch angles from 0° to 179°, the equivalent stiffness matrix can be considered isotropic. When the axis transformation of some layers in the unit volume is 0° and 90°, some components of the stiffness matrix are calculated as zero, as shown in Equation (11).

(11) $${{\bar{C}}_{16}|}_{0{}^{\circ}}={{\bar{C}}_{26}|}_{0{}^{\circ}}={{\bar{C}}_{16}|}_{90{}^{\circ}}={{\bar{C}}_{26}|}_{90{}^{\circ}}=0$$

where the subscript following the vertical bar represents the corresponding hatch angle. Similarly, if θ and −θ exist simultaneously, this can be written as:

(12) $${{\bar{C}}_{16}|}_{\theta }=-{{\bar{C}}_{16}|}_{-\theta }\;{{\bar{C}}_{26}|}_{\theta }=-{{\bar{C}}_{26}|}_{-\theta }$$

Therefore, some components of the equivalent stiffness matrix $\bar{Q}$ are as follows:

(13) $${\bar{Q}}_{16}={\bar{Q}}_{26}=0$$

Equation (6) can be expressed by simply using the following invariant definition [10]:

(14) $$I_{1}=\frac{1}{8}\left(3C_{11}+3C_{22}+2C_{12}+4C_{66}\right)\;I_{2}=\frac{1}{8}\left(C_{11}+C_{22}+6C_{12}-4C_{66}\right)$$

Using the invariant result, an example in which 180 layers are included in a unit volume and the layer rotation is 67° can be written as the following transformed stiffness matrix:

(15) $${\bar{Q}|}_{\sum 67{}^{\circ}\;inc.}={\left[\begin{array}{ccc} {\bar{Q}}_{11} & {\bar{Q}}_{12} & {\bar{Q}}_{16}\\ {\bar{Q}}_{12} & {\bar{Q}}_{22} & {\bar{Q}}_{26}\\ {\bar{Q}}_{16} & {\bar{Q}}_{26} & {\bar{Q}}_{66} \end{array}\right]|}_{\sum 67{}^{\circ}\;inc.}={180\left[\begin{array}{ccc} I_{1} & I_{2} & 0\\ I_{2} & I_{1} & 0\\ 0 & 0 & \frac{I_{1}-I_{2}}{2} \end{array}\right]|}_{\sum 67{}^{\circ}\;inc.}$$

Here, subscript ∑67° inc. denotes the sum of all layers in a unit volume, which is additively manufactured with a hatch angle incremented by 67°. The relationship between each component can be rewritten as follows:

(16) $${\bar{Q}|}_{\sum 67{}^{\circ}\;inc.}={\left[\begin{array}{ccc} {\bar{Q}}_{11} & {\bar{Q}}_{12} & 0\\ {\bar{Q}}_{12} & {\bar{Q}}_{11} & 0\\ 0 & 0 & \frac{{\bar{Q}}_{11}-{\bar{Q}}_{12}}{2} \end{array}\right]|}_{\sum 67{}^{\circ}\;inc.}$$

That is, Equation (16) shows that ${\bar{Q}|}_{\sum 67{}^{\circ}\;inc.}$ is expressed as a stiffness matrix of quasi-isotropic conditions from the rotation of the periodic layer.

## 3. Product Design and AM

### 3.1. Design of the Shock Absorber Mount

**Figure 4:**
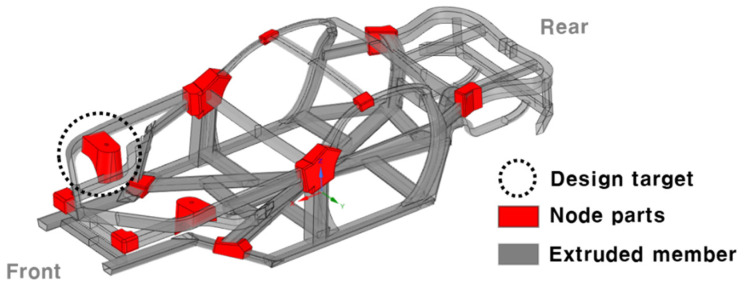
Space frame and node parts of the vehicle.

**Figure 5:**
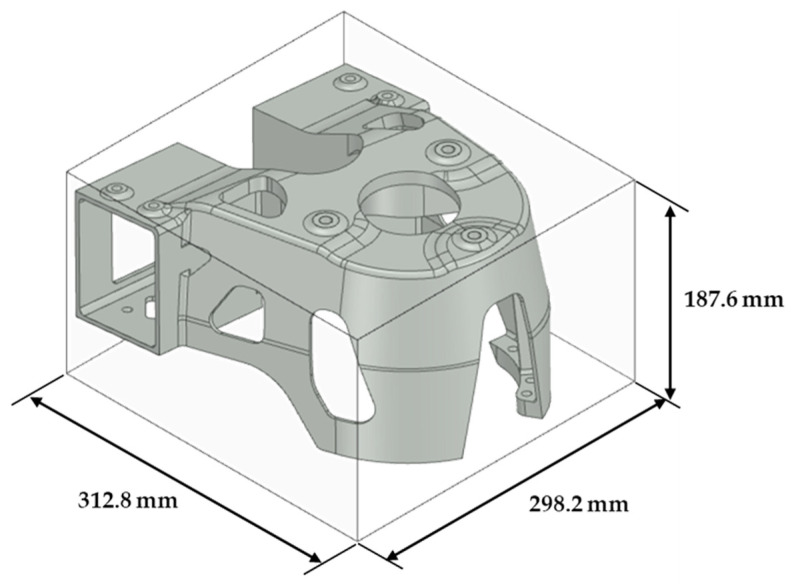
Optimally designed shock absorber node part.

The space frame designed to reduce the weight of a vehicle is composed of an extruded member and the connection of the node parts, as shown in Figure 4. This study designed and manufactured a node part that performs the role of the shock absorber mount. The design process of the node part of the shock absorber mount was described by Yang et al. [15]. In short, the shape of the space frame was derived from the entire vehicle unit, and the concept was designed as a topology optimization for the functional role of the shock absorber mount. The shape was standardized based on the topology optimization result, and the product, as shown in Figure 5, was designed through shape optimization.

### 3.2. AM Process

**Figure 6:**
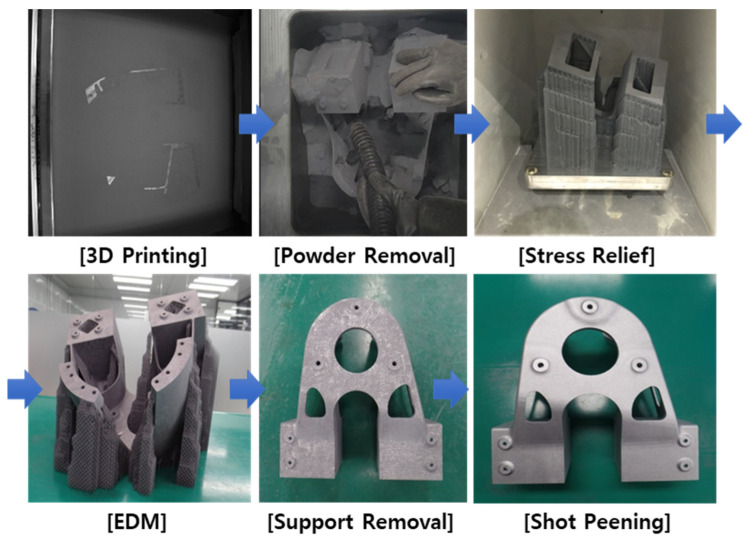
AM process of shock absorber mount node part.

Table 1 lists the AM process parameters applied to the building and the equipment used. AlSi10Mg metal powder, procured from EOS Company (Krailling, Germany), was used as the starting material. In the isotropic analogy of additive-manufactured products using the classical lamination theory in Section 2, the same layer rotation angle (67°) was applied. The layer thickness was 0.03 mm, and if 180 layers were built, the build height would be 5.4 mm. Because the size of the shock absorber mount was sufficiently large, all areas within the entire volume could be considered isotropic. Heat treatment was then performed at 270 °C for 90 min to remove residual stress. Finally, shot peening was performed to ensure a uniform surface roughness. The manufacturing process is illustrated in Figure 6.

## 4. Comparison of the FE Models and Experiments

### 4.1. Material Properties of the FE Model

The results of the FE analysis and experiments on the shock absorber mounts were compared. In the studies by Arisoy et al. [5] and Guan et al. [6], the mechanical properties of a product built through periodic layer rotation were isotropic. According to these previous studies, a periodic rotation strategy at the specimen level was used to suppress the anisotropic properties of the material. However, there are no studies on whether periodic rotation suppresses anisotropy in the case of a large product. Therefore, the present authors intended to manufacture a large product, such as a shock absorber for a vehicle, and evaluate its mechanical properties through experimentation. To confirm whether the consideration of isotropic properties is appropriate, the properties of the FE model were modeled as isotropic and compared with the tensile and modal tests.

**Figure 7:**
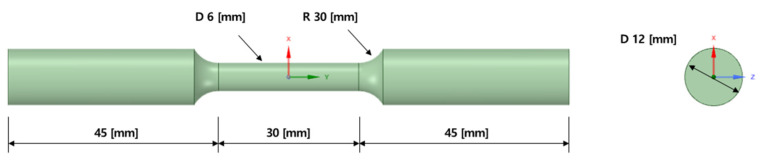
Dimensions of tensile specimens designed to ASTM E8 standards.

First, to obtain the material properties to be reflected in the FE model, a standard tensile specimen test was performed. A dogbone specimen conforming to ASTM E8 was designed [16], as shown in Figure 7, and manufactured under the same AM process conditions as in Table 1.

**Figure 8:**
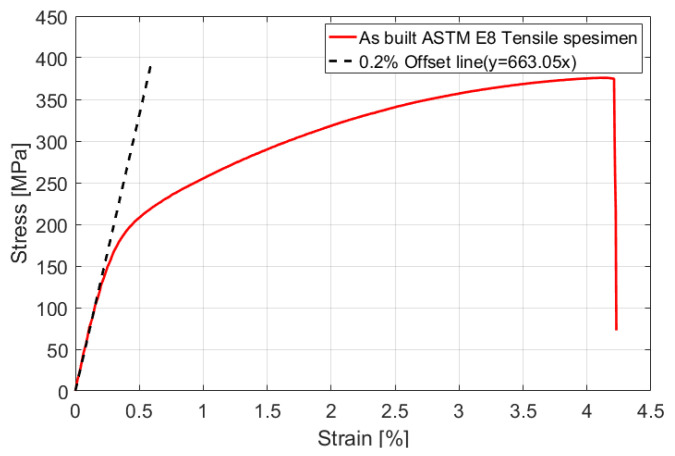
Stress-strain relationship through tensile testing of specimen #1.

The stress-strain curve obtained from the tensile test of the additive-manufactured specimen is shown in Figure 8. The yield strength and elastic modulus of the material were measured using a 0.2% offset, and the results are presented in Table 2. Finally, the tensile test parameters reflected in the equivalent properties of the FE model are listed in Table 3.

### 4.2. Comparison of Tensile Experiment and FE Analysis

A tensile experiment was performed to evaluate the mechanical properties of the manufactured shock absorber mounts. A load of approximately 14 kN was applied while considering the peak value of the Belgian load applied to the front shock absorber mount of the actual vehicle [15]. Accordingly, an appropriate tensile testing machine with sufficient capacity was selected so that even breakage of the designed product could be considered. The test was performed on an Instron tensile tester with a 250 kN specification.

**Figure 9:**
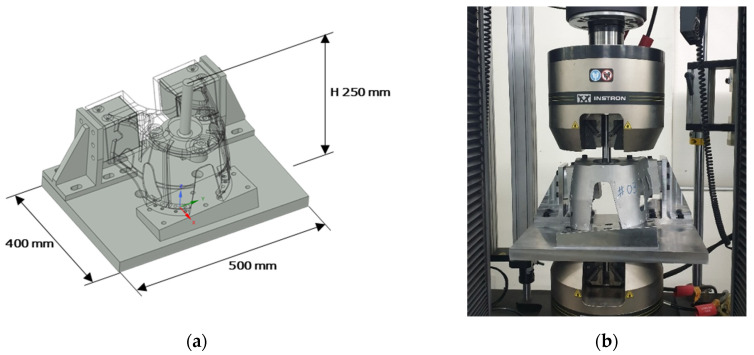
Tensile experiment of shock absorber mount: (**a**) jig design for tensile experiment with dimension; (**b**) shock absorber mount attached to tension equipment.

A tensile test jig, as shown in Figure 9a, which considers the mount position, the extruded part, and the loading condition of the vehicle shock absorber mount, was designed. To apply tension to the shock absorber mount product, a round bar jig was machined on the upper part, and the lower part was machined so that it could be directly bolted to the testing machine. Figure 9b shows the assembled product in the tensile testing machine.

**Figure 10:**
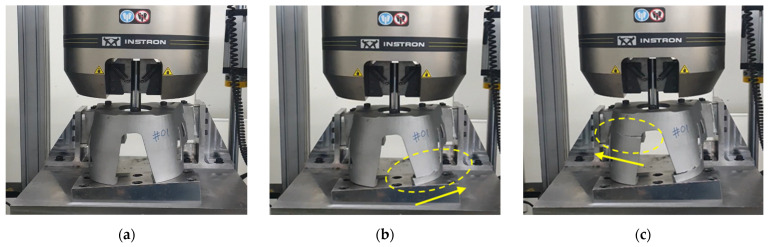
Tensile experiment process: (**a**) the initial stage of tension; (**b**) the stage immediately after the initial fracture; (**c**) the stage of the end of the experiment.

**Figure 11:**
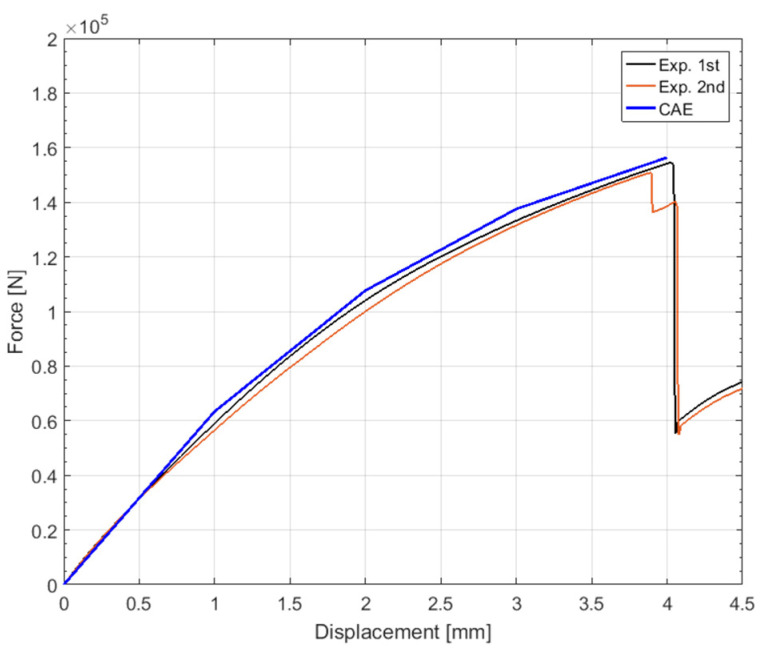
Comparison of load-displacement curve results through tensile test and structural analysis.

To confirm the reproducibility of the experiment, the two products of the fabricated product were tested to fracture at a crosshead speed of 10 mm/min so that the two specimens of the fabricated product were stretched at a sufficiently slow rate for comparison with the static analysis. The tensile experimental procedure and results after the fracture are shown in Figure 10. The location of the fracture and the fracture progress direction are indicated by dotted circles and arrows, respectively. Figure 11 compares the displacement-load curve obtained from the experimental results and the curve obtained from the FE analysis results.

The material properties of the product and the experimental jig were determined to perform an FE analysis of the shock absorber mount. The material properties of the jig and fixing bolts were those of the S45C steel grade, and the properties of the shock absorber mount were those of the additive-manufactured AlSi10Mg powder. The material properties of AlSi10Mg, as determined from the stress-strain curve results obtained from the ASTM-E8 standard test in Section 3.1, were applied to the FE analysis. The values of the material properties of the jigs and bolts applied to the FE analysis model were retrieved from reference [17] and are listed in Table 4.

**Figure 12:**
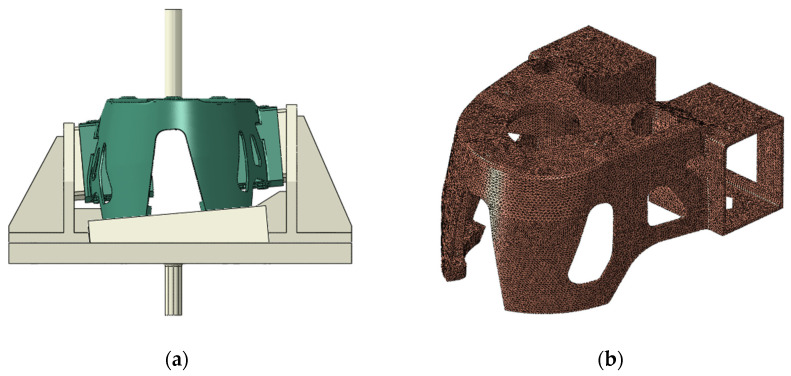
Finite element modeling to perform tensile experiments: (**a**) assembly model; (**b**) finite element model of shock absorber mount.

The FE analysis was performed using ABAQUS, and FE modeling was performed, as shown in Figure 12. The element type used in the analysis model was C3D10M, and a secondary element was applied. The total number of elements in the FE model was 318,584 EA, and the number of nodes was 521,298.

The load-displacement data from the structural analysis are shown together with the experimental results in Figure 11. The results of the maximum load at the fracture of the product are listed in Table 5. The error between the analysis model, which considers the isotropic properties, and the average experimental result is 2.35%, indicating that the analysis and experimental results are in suitable agreement.

**Figure 13:**
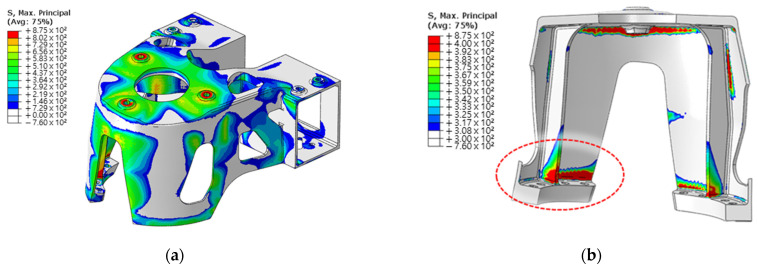
Results of finite element analysis: (**a**) result of maximum stress in the principal axis direction; (**b**) location of stress in excess of tensile strength.

It was possible to predict the stress state and failure location from the results of the FE analysis. Considering that the AlSi10Mg material exhibits brittle properties, the maximum principal axial stress was confirmed in the maximum tensile state, as shown in Figure 13a.

**Figure 14:**
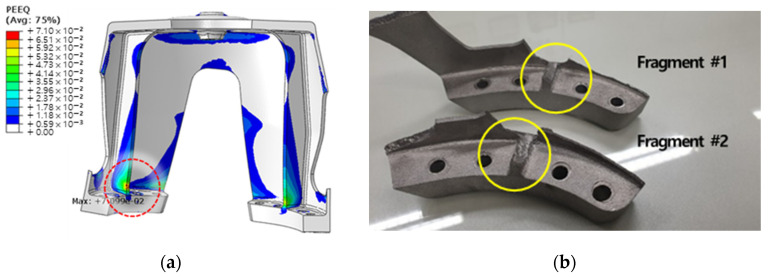
Prediction of the starting location of damage through structural analysis: (**a**) confirmation of high equivalent plastic strain at flange position; (**b**) the location of the failure identified in the fragments of the experiment.

Figure 13b shows that stress exceeding the tensile strength occurs in the inner region of the upper and lower flanges. The equivalent plastic strain results confirmed that the highest occurrence occurred in the inner flange region. Therefore, it was predicted that failure would initiate from the interior of the flange. The fracture locations identified from the experimental procedure and fracture fragments were consistent with those developed from the inside of the flange, as shown in the FE analysis results. The same fracture location was confirmed for the two specimens subjected to tensile testing, as shown in Figure 14.

Consequently, the isotropic material properties applied to the modeling process of the FE analysis did not show any difference from the experimental results, and it was confirmed that the material properties of the products implemented by periodic layer rotation AM could be considered isotropic. This only applies in that case. From the static analysis, as one stiffness value representing the part system is the same in the experiment and FE analysis, the load-displacement curves can appear to match. It may be coincidental that the stiffness of a product that is isotropic or expressed as a combination of anisotropy is the same. Since the coincidence of the initial fracture location is highly dependent on the part shape, fracture may occur at the same location even with different anisotropic properties. For this reason, it cannot be determined as isotropy only by the items analyzed in the static analysis.

### 4.3. Comparison of Modal Test and FE Analysis

Modal tests were performed to evaluate the isotropic properties of the additive-manufactured products. The application of isotropic properties was confirmed by comparing the natural frequencies and modal shapes between the additive-manufactured product and the structural analysis model using the same method as in Section 4.2.

An impact test is applied to the modal test. The response was confirmed at 32 representative locations of the additive-manufactured product using an acceleration sensor. The conditions applied to perform the modal test are listed in Table 6, and the settings applied for the frequency response function (FRF) analysis are listed in Table 7.

**Figure 15:**
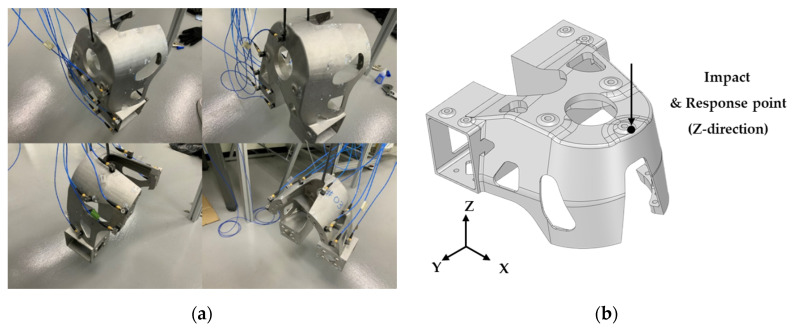
Impact testing process for additive-manufactured product. (**a**) aetting of free-free condition in impact experiment and roving of accelerometer; (**b**) the excitation position of the impact hammer and the position of the acceleration response (z-direction).

The boundary condition of the modal test was a free-free condition, and the product was suspended in air, as shown in Figure 15a, using a rubber band with relatively low stiffness. The locations of the excitation and response are shown in Figure 15b. The impact hammer was used to excite the position marked in the figure in the z-direction, and the response representing the frequency response function was also used in the z-direction at the same position.

In the modal test, the degree of complexity of the modes was confirmed by comparing the modal phase collinearity (MPC). This is a measure of the linear relationship between an imaginary number and the real number of a vector; the more normal the mode is, the closer the result is to 100% [18]. The six natural frequencies are presented in Table 8. Furthermore, the modal similarity was evaluated through a modal assurance criterion (MAC) comparison, and it was confirmed that the six modes of the confirmed natural frequencies were independent [18].

**Figure 16:**
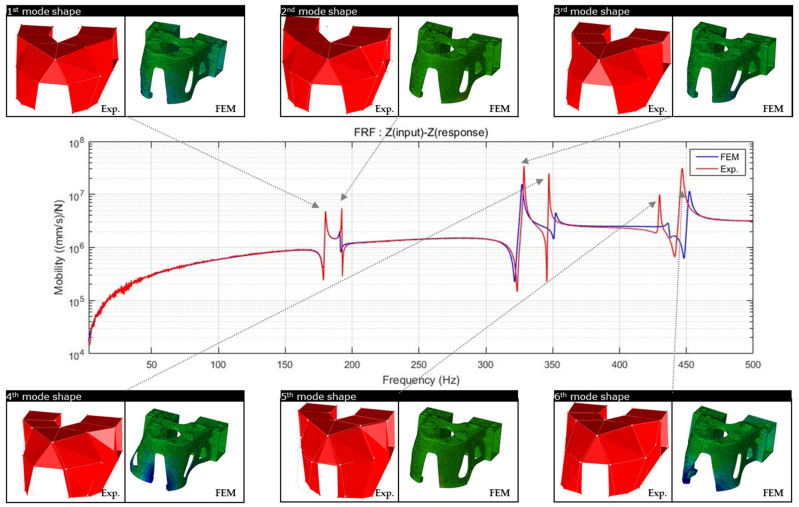
Comparison of natural frequency and mode shape of modal analysis using finite elements and experiment.

The results of the natural frequency measurements obtained from the modal analysis of the FE model are shown in Figure 16.

Modal analysis of the additive-manufactured products through FE analysis was performed in the same manner as described in Section 4.2. The material properties of the product were also considered and modeled as isotropic. Table 9 and Figure 16 show the comparison of the natural frequencies of the FE analysis model and the natural frequency experiments.

The modal shapes of the experimental results and the FE analysis model were highly similar. It was confirmed that the error in the natural frequency results appeared as the higher-order mode ended; however, this was an essential error that appeared because the expression of elements was limited owing to the characteristics of FE modeling. When modeling with a larger number of elements or more than a second-order element, the natural frequency accuracy at higher orders could be better expressed. Consequently, it was confirmed that the results of the modal analysis of the FE model, to which isotropic material properties were applied, and the natural frequency and modal shape of the experiments were in suitable agreement.

## 5. Discussion

As a characteristic of the AM process, anisotropy of macrostructure occurs because of various causes such as the scan direction, melting, fusion, and cooling of the powdered material, and the anisotropy of the material manifests depending on the scanning direction of the laser. A periodic layer rotation strategy was used to suppress the anisotropic properties of the material, which affects the mechanical properties of the product. In this study, the authors aimed to determine whether the periodic layer rotation strategy resulted in isotropic properties, even at the product level. Using the classical lamination theory, it was confirmed that the product fabricated with the periodic layer rotation strategy was isotropically inferred, and the mechanical properties of the additive-manufactured products were evaluated by comparing the stiffness, strength, vibration analysis of the FE model, and experimental results.

The layer-by-layer manufacturing and hatching sequence of the PBF process exhibited the same effect as the layer structure and fiber direction of the composites, and induced anisotropy was present. Therefore, by applying the classical lamination theory to characterize the behavior of the composite, the mechanical properties of the product built using the periodic layer rotation strategy were inferred. As one of the periodic layer rotation strategies, in the case of a hatch angle expressed by a prime number such as 67°, all integer hatch angles were expressed when 180 layers were stacked, which was calculated isotropically according to the equivalent stiffness matrix calculation of the classical lamination theory.

To verify the numerical prediction of isotropic properties through the classical lamination theory at the product level, a shock absorber node, which is part of a vehicle space frame, was manufactured, and tensile and modal tests were performed. The analysis results, which considered the material properties of the FE model to be isotropic, were compared with the results of each experiment. From the tensile tests, the error of the maximum load between the analysis and the test was 2.35%, and it was possible to predict the initial failure location from the results of the FE analysis. The coincidence of the load-displacement curves was confirmed, indicating that the additively manufactured product can be considered isotropic. Importantly, this does not necessarily evaluate to isotropic. With a combination of complex anisotropy, stiffness can be expressed equally. This is because only a single system stiffness was compared for specific directional loads and boundary conditions. Therefore, only the possibility that the material for the product can be considered isotropic is confirmed. Additionally, due to the shape of the part, parts considered anisotropic may also break at the same location.

Additionally, in order to clearly explain the mechanical properties, natural frequency and mode shape analyses were performed to clearly explain this. Products with different anisotropy characteristics, that is, when the scan patterns are different, the natural frequencies are different. From the study of West et al., it was confirmed that the natural frequency was different from the microstructure difference caused by the difference in the scanning strategy [19]. Therefore, it is confirmed that the scan pattern has a sufficient effect on the mechanical properties of the macroscopic structure. In the comparison between the modal test and the FE analysis, the independent first to sixth natural frequency results were compared, and the maximum error (at the fifth natural frequency) was confirmed to be 1.62%. The mode shape analysis shows the shape by considering the mass of the product and the overall and local stiffness values. From the experimental and modal shape analysis of the finite element model, it was confirmed that the expressions of torsion and bending were the same in the deformation behavior of the global and local domains. That is, it was confirmed that the FE analysis, which considered the material properties to be isotropic, was in suitable agreement with the experimental results. Modal analysis is a free vibration analysis for the condition in which there is no damping and external force in the structure, and the eigenvalue problem expressed as a simple harmonic motion has a unique solution, which appears as a result of the natural frequency. Therefore, if the natural frequency of the finite element model considered isotropic and the natural frequency result of the experiment are the same, it means that the product is isotropically built.

In this study, the macrostructure is considered from the product level point of view. Here, depending on the characteristics of the heat flow, the crystal direction of the microstructure may appear, and the anisotropy may appear different from the scan pattern. However, in terms of thermal management and product deformation suppression, the effect is insignificant to consider the thermal characteristics such as the design of the support and the position of the product in the chamber. In addition, the support attached to the large shape of the product level is applied locally, and the thermal properties are reflected only in a very small part of the overall structure. Therefore, microstructure analysis of microstructures according to geometry and support structure that determines heat flow characteristics was excluded. That is, it was judged that the effect would not be significant from the macrostructure point of view. From the previous work of Guan et al. [6] and this study, Experimentation and FE analysis verified that the mechanical properties of the products fabricated by periodic layer rotation AM using the raster scanning method were isotropic. The macrostructural properties of additively manufactured products are anisotropic along the scan direction. When a raster pattern or unidirectional scanning strategy is used in a single layer cross-section, it is expressed as an orthotropic of the macroscopic structure. To simplify and numerically calculate this, the classical lamination theory can be used, and when several layers are overlapped with a hatch angle, it is confirmed that if all directions are expressed with periodicity, it is expressed as isotropy. In addition, as the hatch angle that can express the scan vector in all directions is implemented more isotropic [6], it is necessary to apply the hatch angle with an appropriate period in consideration of all layers. This simple application of the classical lamination theory and the finite element analysis method at the macro level has the advantage of being simpler and faster to apply. Moreover, because the microscopic anisotropy of complex materials was not considered, the FE analysis time was greatly reduced, and applicable results were expected to be obtained.

## 6. Conclusions

1. From a macrostructure point of view, as the anisotropy of an additively manufactured product is induced in the scanning direction, a single layer appears as orthogonal when fabricated with a raster pattern;
2. By applying the classical lamination theory, the overlap of several layers was reflected in the additive manufacturing, and the mechanical properties of the product were evaluated;
3. In the case of additive manufacturing with periodic hatch angles, the mechanical properties of the macrostructure are expressed as isotropy;
4. A tensile test and a modal test were performed to prove that the mechanical properties of the additively manufactured product with a 67-degree hatch angle to which the classical lamination theory is applied are isotropic, and this was verified by comparison with the finite element model;
5. In evaluating the mechanical properties of the macrostructure of additively manufactured products, it is possible to numerically evaluate that the mechanical properties of the product level are isotropically obtained when a periodic hatch angle is applied through the classical stacking theory.

## Figures and Tables

**Table 1:** Laser powder bed fusion process parameters.

Equipment	Parameter	
EOS M400-4	Laser power	370 (W)
	Scan speed	1300 (mm/s)
	Laser diameter	0.1 (mm)
	Hatching distance	0.19 (mm)
	Layer thickness	0.03 (mm)
	Layer rotation angle	67°

**Table 2:** Tensile test result of specimen manufactured by ASTM E8 standard.

Specimen Num.	UTS (MPa)	Yield Strength (MPa)	Strain (%)
# 1	365.87	216.61	4.23
# 2	360.66	223.39	4.30
# 3	363.16	209.69	4.40
Avg.	363.23	216.56	4.31

**Table 3:** Material property data of AlSi10Mg applied to the finite element model.

Material	Elastic Behaviors		Plastic Behaviors	
	Young’s Modulus	Poisson Ratio	True Yield Stress	Plastic Strain
AlSi10Mg (as built)	66305	0.33	98.96	0
			201.64	0.0013530
			327.87	0.0152216
			390.45	0.0353668

**Table 4:** Material property data of jigs and bolts applied to the finite element model.

Material	Elastic Behaviors		Plastic Behaviors	
	Young’s Modulus	Poisson Ratio	True Yield Stress	Plastic Strain
S45C	20,500	0.29	803	0
			904	0.0014833
			1021	0.0049548
			1071	0.0096735
			1103	0.0174457
			1146	0.0336313

**Table 5:** Results of finite element analysis.

Item	Max. Force (kN)	Displacement (mm)
Experiment #1	154.67	4.02
Experiment #2	150.93	3.89
FE Analysis	156.39	4.00

**Table 6:** Methods and equipment for performing modal testing.

**Test equipment**	H/W	LMS SCADAS, 3-axis accelerometer: 10 EA, Impact hammer: 1 EA
	S/W	Simcenter Testlab
**Measurement point**	32 points	
**Method**	Roving accelerometer, Roving direction impact hammer	

**Table 7:** Parameter setting for FRF analysis.

Parameters	Value
Bandwidth	1024
Resolution	0.25 (Hz)
Number of lines	4097
Window type	Exponential (100%)
FRF Estimation	Hv

**Table 8:** Natural frequency result and mode similarity evaluation through modal test.

Mode	Frequency (Hz)	MPC (%)	MAC
1	180.04	85.5	** 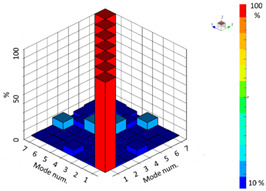 **
2	192.20	84.2	
3	328.45	99.3	
4	347.11	93.2	
5	429.85	97.4	
6	446.70	93.3	

**Table 9:** Comparison of natural frequencies between modal analysis using finite elements and experiments.

Mode	Natural Frequency		Error (%)
	FEA	Experiment	
1	180.02	180.04	0.01
2	190.95	192.20	0.65
3	326.90	328.45	0.47
4	351.88	347.11	1.37
5	436.80	429.85	1.62
6	451.98	446.70	1.18

## Data Availability

The data presented in this study are available on request from the corresponding author and the first author.
